# Two independent functions of Vγ2Vδ2 T cells discriminated by CD16 during HIV-1 infection

**DOI:** 10.1186/1742-4690-9-S2-P169

**Published:** 2012-09-13

**Authors:** X He, H Liang, Y Zhao, H Peng, D Liu, Y Shao

**Affiliations:** 1 National Center for AIDS/STD Control and Prevention, China CDC, Beijing, China

## Background

Vγ2Vδ2 (Vδ2) T cells play a vital role in the control of HIV infection. Vδ2 T cells recognize phosphoantigens such as IPP, and they mediate ADCC through FcγRIIIa (CD16). Our goal is to understand how the heterogeneous repertoires of Vδ2 T cells are involved in both phosphoantigen -induced response and ADCC in HIV infection, especially in the early stage of HIV infection.

## Methods

PBMCs were obtained from a total of 81 subjects, including 18 early, 42 chronic HIV-1 infected subjects (all treatment-naive) and 21 healthy subjects. Cellular immune functions of Vδ2 T cells were analyzed by flow cytometry.

## Results

Circulating Vδ2 T cells comprised two functionally diverse subsets which were discriminated by the CD16 expression. Most cytotoxic molecules and IFN-γ were released by CD16^-^ subset (98% in average) after IPP stimulation, while the CD16^+^ subset was in charge of triggering ADCC via CD16 that was closely related to HIV-associated changes in Vδ2 T cell-mediated ADCC (p< 0.001). In early HIV infection, the CD16^-^ Vδ2 T cells dramatically decreased in comparison with healthy controls (p=0.02), accompanied by the decline of IPP-responsive Vδ2 T cells (p=0.01). Interestingly, a dramatic functional switch of Vδ2 T cell-mediated ADCC with almost reverse profile of the CD107a and IFN-γ expression compared to uninfected group was observed since early HIV infection. Frequency of CD107a+ Vδ2 T cells from early-infected group was significantly higher than that from healthy controls (p<0.05). Although the IPP-activated Vδ2 T cells declined notably in chronic-infected individuals with CD4>500 (cells/μl), the percentage of antibody-dependent cytotoxic Vδ2 T cells was over threefold as high in CD4>500 individuals as in healthy controls (p<0.05 for both).

## Conclusion

These data revealed the involvement of two Vδ2 T subsets with different functions during HIV infection and highlighted the plasticity of Vδ2 T cell-mediated ADCC in controlling HIV infection.

